# Editorial: Early detection, cancer interception, et al.: translating the multifaceted use of liquid biopsy to the management of early disease

**DOI:** 10.3389/fonc.2026.1879673

**Published:** 2026-05-27

**Authors:** Letizia Pontolillo, Lorenzo Gerratana, Carolina Reduzzi

**Affiliations:** 1 Department of Medicine, Division of Hematology-Oncology, Weill Cornell Medicine, New York, NY, United States; 2 Department of Translational Medicine and Surgery, Università Cattolica del Sacro Cuore, Rome, Italy; 3 Department of Medical Oncology, Centro di Riferimento Oncologico di Aviano (CRO), National Cancer Institute, Istituto di Ricovero e Cura a Carattere Scientifico (IRCCS), Aviano, Italy; 4 Department of Medicine, University of Udine, Udine, Italy

**Keywords:** cell-free DNA, circulating tumor DNA (ctDNA), early detection, liquid biopsy, minimal residual disease (MRD)

Early detection remains one of the most critical determinants of cancer outcomes. Despite significant advances in imaging and systemic therapies, a substantial proportion of patients diagnosed with localized disease ultimately develop metastatic relapse, often years after initial treatment. Traditional radiologic surveillance enables the detection of clinically overt recurrence, yet it lacks the sensitivity to capture subclinical disease. In this context, the emergence of liquid biopsy has introduced a paradigm shift, enabling the identification of tumor-derived signals in body fluids and fostering the concept of minimal residual disease (MRD) as a measurable and actionable entity.

Circulating tumor DNA (ctDNA) has become the most widely studied analyte for MRD detection, allowing the identification of molecular relapse before radiographic progression. However, the clinical implications of MRD detection remain incompletely defined, particularly regarding whether therapeutic intervention based on molecular relapse translates into improved survival (1). Beyond ctDNA, other circulating components—including circulating tumor cells (CTCs), cell-free RNA, proteins, and extracellular vesicles—are increasingly being investigated, expanding the framework toward a more comprehensive and multifaceted approach. Concepts such as cellular residual disease (CRD) further emphasize the need to integrate multiple biomarkers to better capture tumor heterogeneity and disease dynamics (2).

This Research Topic was conceived to provide a comprehensive overview of how liquid biopsy can be translated into the management of early-stage disease, spanning early detection, MRD assessment, and cancer interception. The Research Topic highlights both technological advances and clinical challenges, illustrating the evolving role of liquid biopsy across different tumor types and clinical contexts.

A first group of contributions focuses on biomarker discovery and early detection strategies, emphasizing the diversity of analytes and platforms under investigation. Jiang et al. identified a serum three- long noncoding RNA (lncRNA) panel with high diagnostic accuracy for muscle-invasive bladder cancer, supporting the promising role of transcriptomic profiling as a minimally invasive diagnostic tool.

In their systematic review, Romero Urrego et al. provide an overview of the growing relevance of circulating microRNAs, derived from both extracellular vesicles and non-extracellular vesicles sources, as promising biomarkers for early breast cancer detection, with several candidate miRNAs and panels demonstrating high diagnostic accuracy in distinguishing breast cancer cases from health controls. Together, these studies underscore the potential of microRNA-based biomarkers to complement and potentially enhance current screening approaches.

The expansion of multi-cancer early detection (MCED) approaches represents another major theme within the Research Topic. Walter et al. evaluated an innovative screening blood-based test (Carcimun^®^), capable of detecting conformational changes in plasma proteins through optical extinction measurements. It showed a high sensitivity and specificity in distinguishing cancer patients from healthy individuals and subjects with inflammatory conditions, emphasizing the importance of reducing false-positive results in real-world clinical settings. Choudhry et al. in an exploratory analysis of the DETECT-A study further explored the clinical implications of positive MCED testing, demonstrating that these approaches may also uncover clinically significant pre-malignant lesions amenable to curative intervention. These findings reinforce the emerging concept of cancer interception, in which molecular abnormalities can be identified before the development of overt invasive disease.

A second major area of interest concerns the role of liquid biopsy in MRD detection and disease monitoring. Appierto et al. demonstrated in the prospective phase III fenretinide prevention trial that ctDNA detection could anticipate loco-regional recurrence in early-stage breast cancer up to 28 months before clinical diagnosis, supporting its potential role in follow-up strategies and risk stratification. Similarly, Wang et al. reported a case of locally advanced hypopharyngeal carcinoma in which persistent ctDNA positivity during neoadjuvant treatment revealed occult molecular progression despite a favorable radiologic response. This case highlights the limitations of imaging alone in assessing treatment response and further supports the integration of molecular monitoring into clinical decision-making.

The Research Topic also illustrates the broad applicability of liquid biopsy across multiple tumor types and biological specimens. Feng et al. reviewed the clinical utility of different liquid biopsy samples for ovarian cancer early detection, including plasma, urine, uterine lavage, and cervicovaginal specimens, emphasizing the growing role of multi-omics approaches in improving diagnostic accuracy. Similarly, Wang et al. demonstrated the feasibility of detecting cell-free DNA from both peripheral blood and Papanicolaou smears in endometrial cancer, suggesting that minimally invasive gynecologic sampling may complement blood-based and tissue assays for early diagnosis and risk stratification.

Another emerging field highlighted in this Research Topic is the potential role of liquid biopsy in guiding therapeutic escalation and de-escalation strategies. Hollander and Nonaka conducted a metanalysis showing the high diagnostic performance of cell-free HPV-DNA in HPV-positive head and neck squamous cell carcinoma, supporting its possible use as a biomarker to guide treatment de-intensification approaches. In parallel, Kravchuk et al. described the dynamic assessment of blood microsatellite instability during immunotherapy in a patient diagnosed with colorectal cancer, suggesting that longitudinal liquid biopsy monitoring may provide clinically relevant information on treatment response and hyperprogressive disease.

Finally, disease-specific translational perspectives further contextualize the integration of liquid biopsy into early cancer management. Cox et al. reviewed the multifaceted role of liquid biopsy in pancreatic ductal adenocarcinoma, discussing applications ranging from early detection and molecular subtyping to treatment monitoring and MRD assessment. Importantly, many contributions across this Research Topic consistently emphasize the need for assay standardization, prospective validation, and better definition of clinically actionable thresholds before widespread implementation into routine practice can be achieved.

Taken together, the articles included in this Research Topic portray a rapidly evolving field that is progressively moving from proof-of-concept studies toward clinical translation. Nevertheless, several challenges remain unresolved. The optimal integration of MRD testing into clinical workflows, the interpretation of persistent molecular positivity, the timing of sample collection, and the identification of patients who may benefit from treatment escalation or de-escalation still require prospective validation through dedicated clinical trials.

Despite these limitations, liquid biopsy holds enormous promise to reshape cancer management by enabling earlier detection, more precise disease monitoring, and timely therapeutic intervention. The transition from reactive to proactive oncology, where disease can potentially be intercepted before clinical manifestation, represents one of the most important paradigms shifts in modern cancer care.

In conclusion, this Research Topic provides a comprehensive and timely overview of the multifaceted role of liquid biopsy in early cancer management. By integrating advances in biomarker discovery, technological innovation, and clinical application, the studies collected here highlight both the remarkable opportunities and the critical challenges that lie ahead in translating liquid biopsy into routine clinical practice.
